# Dehiscence of detached internal limiting membrane in eyes with myopic traction maculopathy with spontaneous resolution

**DOI:** 10.1186/1471-2415-14-39

**Published:** 2014-03-29

**Authors:** Kazunari Hirota, Akito Hirakata, Makoto Inoue

**Affiliations:** 1 Kyorin Eye Center, Kyorin University School of Medicine, 6-20-2 Shinkawa, Mitaka, Tokyo 181-8611, Japan

**Keywords:** Optical coherence tomography, Myopic traction maculopathy, Myopia, Retinoschisis

## Abstract

**Background:**

To report the optical coherence tomographic (OCT) findings in 4 eyes before and after a spontaneous resolution of a myopic traction maculopathy (MTM).

**Method:**

Retrospective review of medical records including history, examination findings, fundus details, and finding of spectral-domain OCT (Cirrus HD-OCT, Carl Zeiss Meditec, Spectralis, Heidelberg Engineering, Heidelberg) findings in 4 eyes with a spontaneous resolution of MTM.

**Results:**

A release of the vitreofoveal traction was detected by OCT in 3 eyes before the resolution of the MTM. A vitreofoveal separation in one eye and an increase in the length of a vitreous strand from the macula in two eyes indicated a reduction in the traction. In 2 eyes, an internal limiting membrane (ILM) detachment was seen by OCT as a membrane above the wrinkled inner retina at the perifoveal lesion, and a flattening of the ILM and inner retina was detected after the resolution. The detached ILM was shifted centrifugally on the macula and disappeared with the flattening of the adjacent retina which suggests that the release of tangential traction was caused by the dehiscence of ILM possibly at the proximal edges of the ILM detachment.

**Conclusion:**

Releasing a vitreofoveal traction and flattening of the detached ILM may be signs of spontaneous resolution of a MTM. Vitrectomy is not required when these signs are detected.

## Background

Myopic traction maculopathy (MTM) occurs in 9% of highly myopic eyes with a posterior staphyloma and causes a reduction in vision [1]. Anteroposterior or tangential traction by the vitreous cortex on the internal limiting membrane (ILM) and retinal vessels has been suggested to be a major cause of MTM [2,3]. However, there are some cases of MTM that resolve spontaneously without surgery [4]. We report the optical coherence tomographic (OCT) findings of eyes before and after a spontaneous resolution of MTM.

## Methods

This was a retrospective study of four cases of MTM which resolved spontaneously (Table 1). Written informed consent was obtained from the patient for publication of these case reports and any accompanying images. A copy of the written consent is available for review by the Editor of this journal. The best-corrected visual acuity (BCVA), ophthalmoscopic findings, and OCT findings (Cirrus HD-OCT Model 4000, Carl Zeiss Meditec Inc, Dublin, CA, Spectralis, Heidelberg Engineering, Heidelberg) were evaluated.

**Table 1:** Clinical characteristics of eyes with myopic tractional myopathy

**Case**	**Age**	**Sex**	**Eye**	**BCVA**	**Lens**	**Refractive errors (D)**	**Axial length (mm)**	**Vitreous separation**	**ILM detachment**
1	57	M	L	0.8	Phakic	−7.5	27.66	+	-
2	68	F	R	0.4	IOL	−6.5	29.52	+	+
3	88	F	R	0.2	Phakic	−9.5	28.36	-	+
4	41	F	R	1.0	Phakic	−9.5	27.02	+	-

BCVA = best-corrected visual acuity, D = diopter, ILM = internal limiting membrane, F = female, M = male, L = left, R = right, IOL = intraocular lens.

## Results

### Case reports

#### Case 1

**Figure 1:**
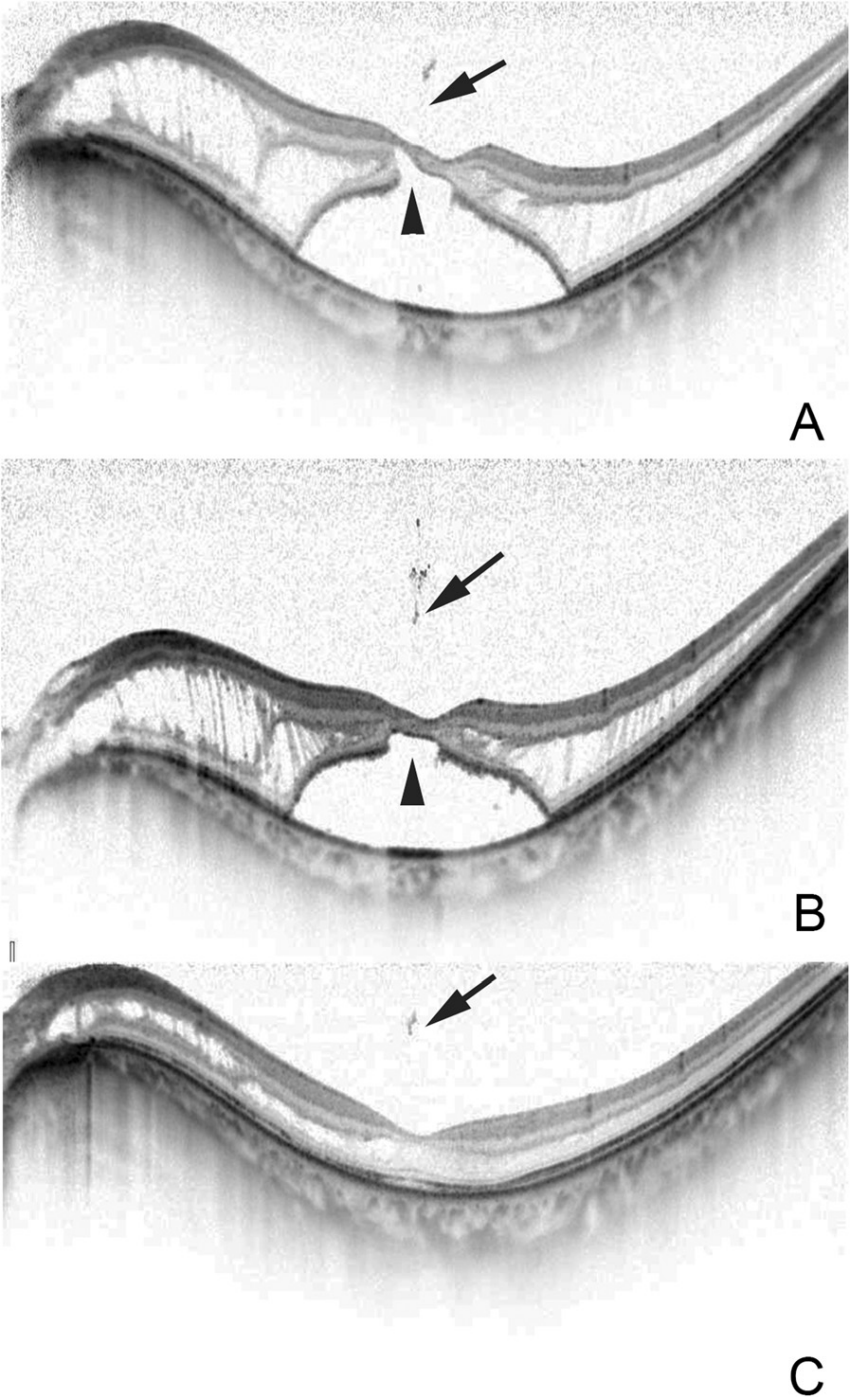
**Optical coherence tomographic (OCT) images of Case 1. A**: OCT image showing macular retinoschisis and foveal detachment. A vitreous strand (arrow) from a vitreous opacity is attached to the retinal surface. An outer retinal break (arrowhead) can be seen beneath the vitreous strand. **B**: After 2 months, the macular retinoschisis and foveal detachment remain but the vitreous strand (arrow) is detached and farther away from the retina. The outer retinal break is sealed (arrowhead). **C**: After 3 months, the foveal detachment is completely resolved with partial macular retinoschisis but the vitreous strand remains (arrow).

A 57-year-old man noticed a blurring of vision and metamorphopsia in his left eye. His decimal BCVA was 0.8, and OCT showed a macular retinoschisis with foveal detachment in the left eye (Figure 1). A vitreous strand was seen attached to the parafoveal lesion, and an outer retinal break was identified beneath the point of attachment and the vitreous surgery was scheduled. Two months later, the BCVA improved to 0.9 with a resolution of the metamorphopsia, and we decided to follow the patient without surgery.

OCT examination showed a detachment of the vitreous strand and the outer retinal break was sealed. Three months later, the foveal detachment was flattened with a decrease of the macular retinoschisis as confirmed by OCT.

#### Case 2

**Figure 2:**
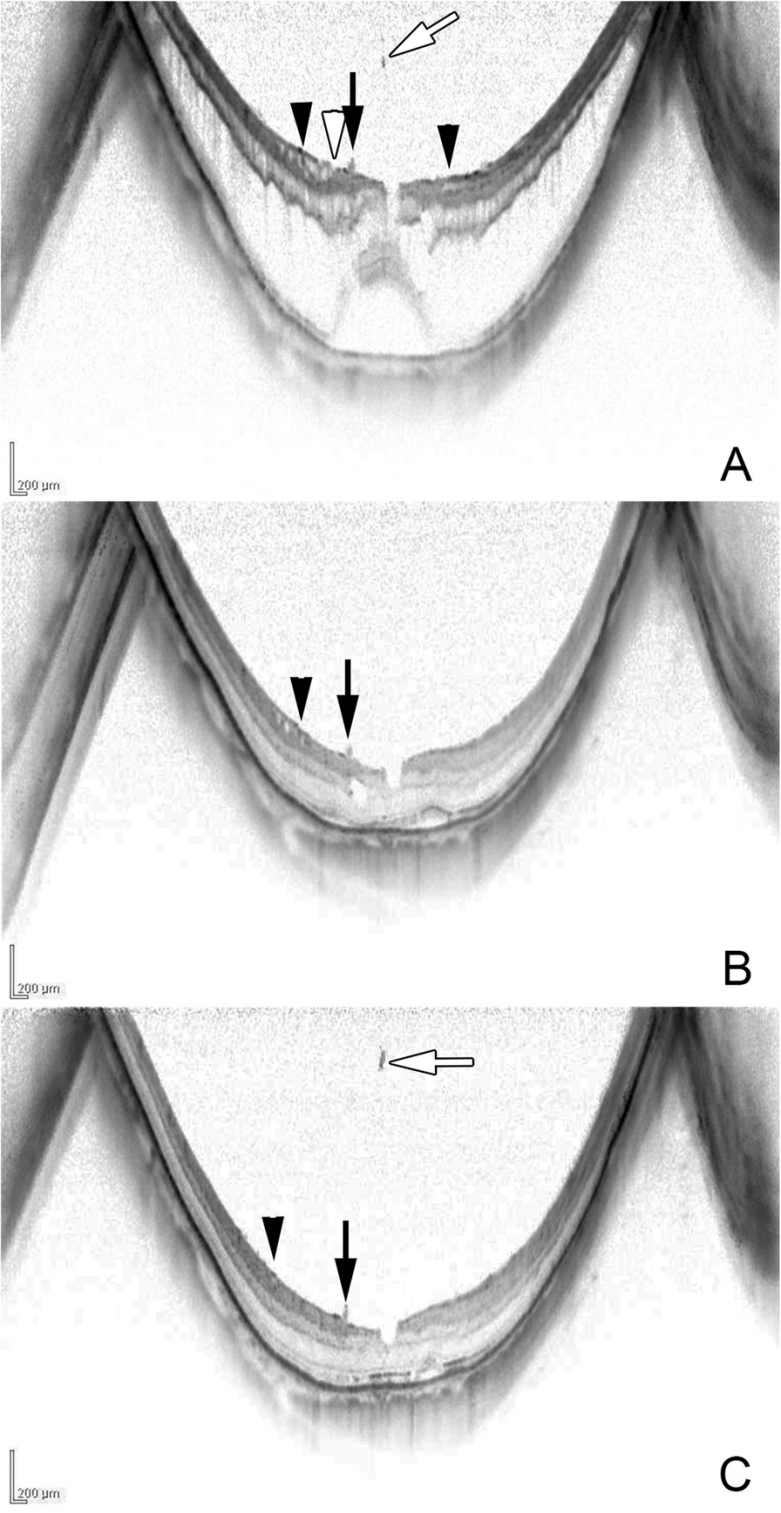
**Optical coherence tomographic (OCT) images of Case 2. A**: OCT image shows macular retinoschisis and foveal detachment with vitreous strand (white arrow) attached at the parafovea (arrow). Tractional internal limiting membrane (ILM) detachment (arrowhead) is detected as a membrane above the wrinkled inner retina at the perifoveal lesion. ILM dehiscence (white arrowhead) is also seen. Vitreous strand (arrow) from the vitreous opacity is attached to the retinal surface. **B**: After one month, the macular retinoschisis and foveal detachment are decreased and the tractional ILM detachment is also decreased and shifted centrifugally to the temporal side (arrowhead). The vitreous strand remains attached (arrow). **C**: After 3 months, macular retinoschisis and tractional ILM detachment (arrowheads) are completely resolved, and the wrinkled inner retina is flattened. However, the vitreous strand (arrow) remains with increasing distance from the vitreous opacity (white arrow).

A 68-year-old woman noted a decrease in her vision with metamorphopsia in her right eye. OCT examination showed macular retinoschisis and foveal detachment with a membrane at the perifoveal lesion connected to the retina. The membrane was most likely the ILM detached from the retina (Figure 2). A vitreous strand was attached at the parafoveal region beside the ILM detachment. One month later, the BCVA improved to 0.8 with a resolution of the metamorphopsia. OCT examination showed a resolution of the macular retinoschisis and foveal detachment. The ILM detachment decreased and shifted to the temporal side although the vitreous strand was still attached to the parafoveal lesion. Three months later, the macular retinoschisis was resolved but the ILM detachment remained flattened, and the vitreous opacity was located farther from the attachment of the vitreous strand.

#### Case 3

**Figure 3:**
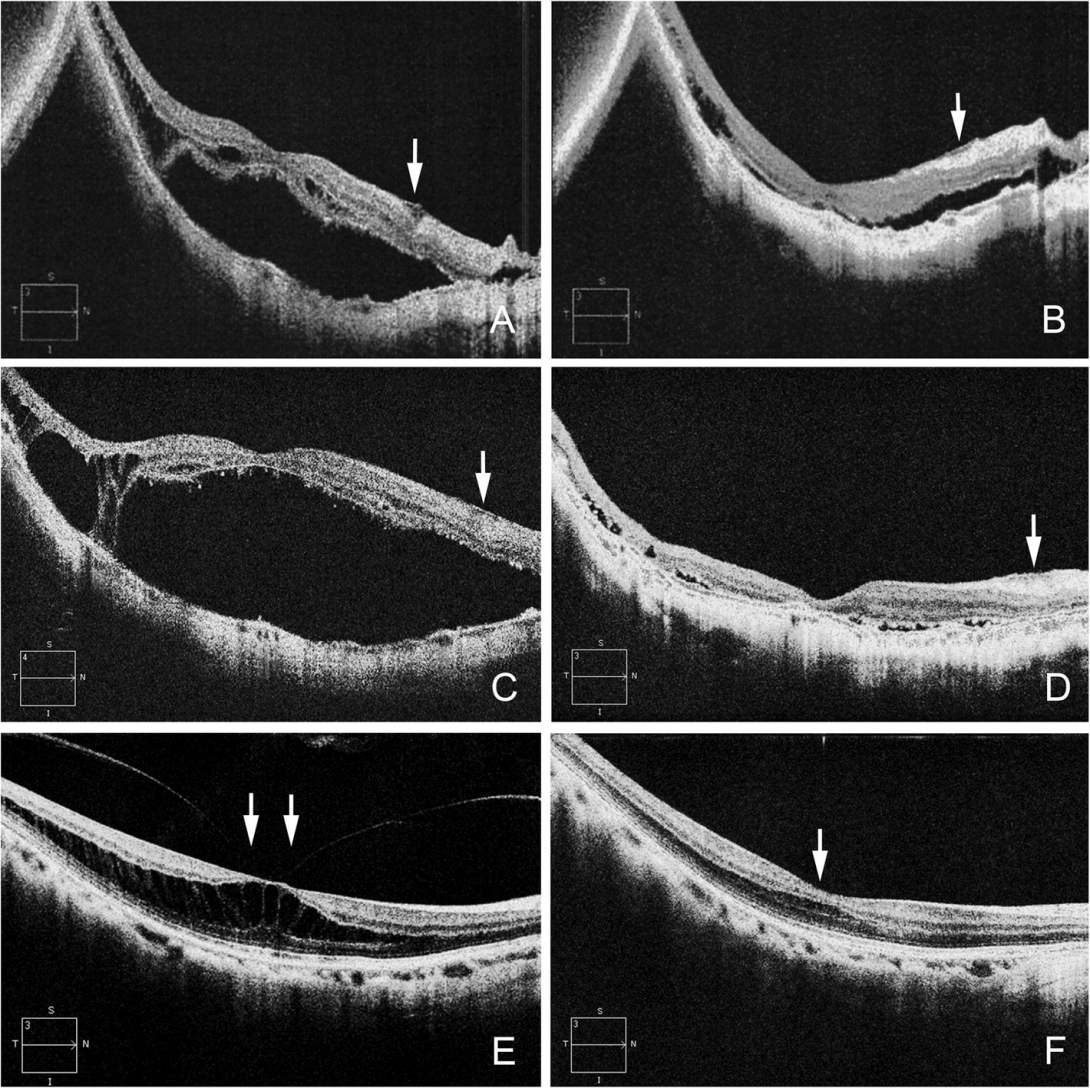
**Optical coherence tomographic (OCT) images of Cases 3 and 4. A**: OCT images of Case 3 showing macular retinoschisis and foveal detachment with internal limiting membrane detachment (white arrow) between the macula and the optic disc. **B**: After one month, the macular retinoschisis and foveal detachment decrease with a decrease of the ILM detachment (white arrow). **C**: After 10 months, macular retinoschisis and foveal detachment recur due to a paravascular retinal break at the inferior vascular arcade but the ILM detachment remains flattened (white arrow). **D**: The retina is reattached after vitreous surgery with resolution of the ILM detachment (white arrow). **E**: OCT images of Case 4 showing macular retinoschisis with parafoveal posterior vitreous detachment (white arrows). **F**: After 12 months, macular retinoschisis has resolved (white arrow) after vitreofoveal separation.

An 88-year-old woman reported visual difficulties and metamorphopsia in her right eye. Her BCVA was 0.2, and OCT examination showed macular retinoschisis and foveal detachment with a detachment of the ILM between the macula and the optic disc (Figure 3). Eleven months later, the degree of metamorphopsia was reduced but the BCVA remained at 0.2. Fundus examination disclosed a glial ring, and OCT examination showed a decrease in the macular retinoschisis and foveal detachment. The ILM detachment flattened and the inner retina beneath the ILM detachment also became flatter. Two years later, she noticed a sudden visual reduction with her BCVA reduced to 0.15. Fundus and OCT examinations revealed a recurrence of the MTM. The patient underwent vitrectomy, and a complete posterior vitreous detachment was confirmed intraoperatively, and a paravascular retinal break at the inferior vascular arcade and at the edge of a chorioretinal atrophic lesion temporal to macula was identified. These were considered to be the causes of the recurrence of the MTM. After vitrectomy, the retina was successfully reattached and vision recovered to 0.2. OCT confirmed the reattachment of the retina and the ILM detachment remaining flattened.

#### Case 4

A 41-year-old woman reported visual disturbances and metamorphopsia in her right eye. Her BCVA was 1.0 but OCT examination showed macular retinoschisis with a parafoveal posterior vitreous detachment pulling on the macula (Figure 3). Twelve months later, a vitreofoveal separation developed and the macular retinoschisis spontaneously resolved. The BCVA improved to 1.2.

## Discussion

The release of a vitreofoveal anteroposterior traction in 3 of the 4 eyes led to the resolution of the MTM. A vitreofoveal separation in one eye and an increase in the length of a vitreous strand from the macula in two eyes suggested a reduction in the traction. However, the vitreous opacity did not remain in the same place, and the length of the vitreous strand may not remain constant. This sign of the length of the vitreous strand from the macula needs to be evaluated more precisely although a vitreous separation in Case 4 was apparent. Tractional ILM detachments have been reported in highly myopic eyes with posterior staphylomas, and the rigidity of the ILM has been suggested to cause the myopic retinoschisis [5]. In 2 of 4 eyes, an ILM detachment was seen by OCT as a membrane above the wrinkled inner retina at the perifoveal lesion, and a flattening of the ILM detachment and the inner retina was detected after the resolution. The detached ILM was shifted to the temporal side in Case 2 and to the nasal side in Case 3. Both directions would cause centrifugal tension on the macula, a disappearance of the detached ILM. The flattening of the adjacent retina suggested that the release of tangential traction and spontaneous resolution of MTM were caused by the separation of the ILM possibly at the proximal side of the ILM detachment. Shimada and associates [6] reported that 8 of 207 eyes (3.9%) had a decrease or complete resolution of the macular retinoschisis after a posterior vitreous detachment or a spontaneous disruption of the ILM during a mean follow-up of 36.2 ± 6.2 months.

We believe that the dehiscence of the edges of the detached ILM is a sign of a release of traction. In Case 3, the retinal reattachment that occurred following the release of the ILM traction may have caused the paravascular retinal tears where the flexibility of retina is reduced by the retinal vessel. Our findings indicate that releasing the vitreofoveal traction and also flattening of detached ILM may be signs of spontaneous resolution of the MTM.

## Conclusions

Releasing vitreofoveal traction and flattening of the detached ILM may be signs of spontaneous resolution of the MTM. Vitrectomy is not required when these signs of dynamic change around the vitreoretinal interface are detected.

## Abbreviations

MTM: Myopic traction maculopathy; ILM: Internal limiting membrane; OCT: Optical coherence tomography; BCVA: Best-corrected visual acuity.

## Competing interests

Non-financial competing interests.

## Authors’ contributions

Involved in management, analysis, interpretation, and preparation of the data (KH, MI, AH). Involved in interpretation, and preparation of the manuscript (KH, MI, AH). All authors read and approved the final manuscript.

## Pre-publication history

The pre-publication history for this paper can be accessed here:

http://www.biomedcentral.com/1471-2415/14/39/prepub

## References

[B1] Baba T Ohno-Matsui K Futagami S Yoshida T Yasuzumi K Kojima A Tokoro T Mochizuki M Prevalence and characteristics of foveal retinal detachment without macular hole in high myopia Am J Ophthalmol 2003 135 338 342 10.1016/S0002-9394(02)01937-2 12614751

[B2] Ikuno Y Sayanagi K Soga K Oshima Y Ohji M Tano Y Foveal anatomical status and surgical results in vitrectomy for myopic foveoschisis Jpn J Ophthalmol 2008 52 269 276 10.1007/s10384-008-0544-8 18773264

[B3] Gaucher D Haouchine B Tadayoni R Massin P Erginay A Benhamou N Gaudric A Long-term follow-up of high myopic foveoschisis: natural course and surgical outcome Am J Ophthalmol 2007 143 455 462 10.1016/j.ajo.2006.10.053 17222382

[B4] Polito A Lanzetta P Del Borrello M Bandello F Spontaneous resolution of a shallow detachment of the macula in a highly myopic eye Am J Ophthalmol 2003 135 546 547 10.1016/S0002-9394(02)02080-9 12654378

[B5] Sayanagi K Ikuno Y Tano Y Tractional internal limiting membrane detachment in highly myopic eyes Am J Ophthalmol 2006 142 850 852 10.1016/j.ajo.2006.05.031 17056366

[B6] Shimada N Tanaka Y Tokoro T Ohno-Matsui K Natural course of myopic traction maculopathy and factors associated with progression or resolution Am J Ophthalmol 2013 156 948 957 10.1016/j.ajo.2013.06.031 23972301

